# Novel clinical indenoisoquinoline topoisomerase I inhibitors: a twist around the camptothecins

**DOI:** 10.18632/oncotarget.26466

**Published:** 2018-12-18

**Authors:** Yves Pommier, Mark Cushman, James H. Doroshow

**Affiliations:** Yves Pommier: Developmental Therapeutics Branch and Laboratory of Molecular Pharmacology, Center for Cancer Research, Division of Cancer Treatment and Diagnosis, National Cancer Institute, NIH, Bethesda, Maryland, USA

**Keywords:** topoisomerase, therapeutics

Topotecan and irinotecan are both derivatives of the plant alkaloid camptothecin [1]. They are widely used for the treatment of solid tumors including colon, lung and ovarian cancers as well as pediatric tumors. Since their FDA approval almost 20 years ago, they have remained the only marketed topoisomerase I (TOP1) inhibitors [2]. This situation is unique in the anticancer armamentarium and is primarily related to the fact that pharmaceutical companies turned to the discovery of protein kinase inhibitors (often referred to as “targeted therapies”) shortly before the clinical approval of topotecan and irinotecan.

**Figure 1:**
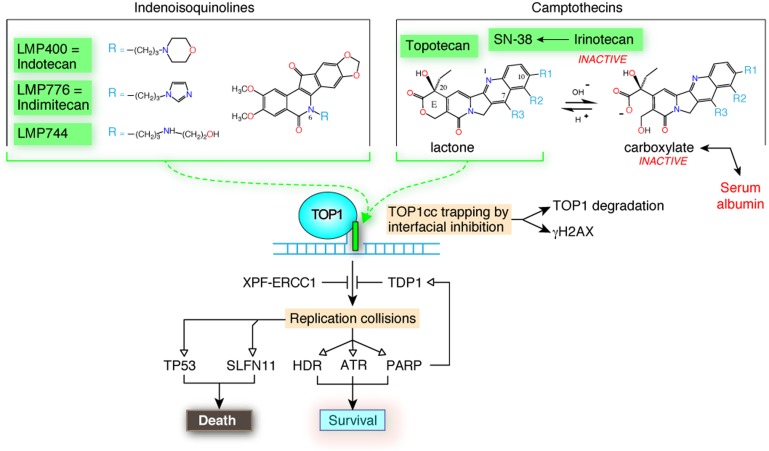
Clinical TOP1 inhibitors and cellular determinants of response The chemical structures of the clinical indenoisoquinoline derivatives, LMP400, LMP776 and LMP744 are shown at the top left. The camptothecin derivatives are shown at the right. Camptothecins are active in the lactone form and are readily inactivated at physiological pH in the blood and tissues by E-ring hydrolysis to the ring-open carboxylate form, which is sequestered by albumin. Middle: both the indenoisoquinolines and camptothecins trap TOP1 cleavage complexes (TOP1cc) by binding at the enzyme-DNA interface. Trapped TOP1cc induce the rapid degradation of TOP1 by the ubiquitin proteasome pathway and engage the chromatin response by phosphorylation of histone H2AX (γH2AX). TOP1cc are excised by TDP1 (tyrosyl DNA phosphodiesterase) and the endonuclease XPF-ERCC1. The primary cytotoxic lesions in cancer cells result from collisions between the trapped TOP1cc and replication forks. These collisions are repaired by engaging HDR (homology directed repair) and activating ATR (Ataxia Telangiectasia related) kinase and PARP (poly[ADPribose]polymerase). Replication collisions also activate the cell death pathways by engaging p53 (TP53) and Schlafen 11 (SLFN11).

Yet, camptothecins can be nominated “targeted therapies” as they only target TOP1, and crystal structures demonstrate that only the active natural stereoisomers (20-S) of the camptothecins (see top right of the Figure 1) bind to the TOP1-DNA interface (scheme in the middle of the Figure 1), stacking with the base pairs that flank the DNA cleavage site generated by TOP1 and forming a network of hydrogen bonds with amino acid residues of the TOP1 catalytic pocket [3]. This finding led to the concept of interfacial inhibition, a molecular mechanism of action for a wide variety of natural products including TOP2 inhibitors, rapamycin, tubulin inhibitors and anesthetic drugs [3]. Binding of a single molecule of a camptothecin or indenoisoquinoline [4] traps the TOP1-DNA complex by blocking the religation of the broken DNA and the release of TOP1, which remains covalently attached to the 3’-end of the break that TOP1 made to relax DNA. As a result, camptothecins and indenoisoquinolines act by producing TOP1 cleavage complexes (TOP1cc).

In spite of their demonstrated clinical activity, camptothecins have limitations : 1) chemical instability that converts the active lactone E-ring to a carboxylate, which is sequestered by binding to serum albumin (top right panel); 2) irinotecan is a prodrug requiring its conversion by carboxylesterase (primarily in the liver) to SN-38, its active metabolite; 3) camptothecins are substrates for drug efflux transporters (primarily ABCG2); 4) the serum half-life of camptothecin lactones is short (2-4 hours); and 5) irinotecan can produce severe diarrhea due to its biliary elimination and activation by the microbiome.

We embarked on the discovery of non-camptothecin TOP1 inhibitors to overcome the limitations of camptothecins [5]. Also, because camptothecins are the only class of TOP1 inhibitors, development of another chemical class might produce molecules that exhibit distinct properties; for instance, colchicine and vinblastine both poison tubulin polymerization but have different medical applications. The first indenoisoquinoline candidate TOP1 inhibitor was discovered using the large drug and NCI-60 database of the Developmental Therapeutics Branch of the NCI. Because many camptothecin derivatives had been tested, we performed a pattern comparison analysis to identify drug candidates having the characteristic activity of the camptothecins while being chemically unrelated. Out of the hundreds of thousands of compounds in the database, our previously synthesized indenoisoquinoline, NSC314622 [6] was identified. Analogs were generated and optimized to improve the potency of the indenoisoquinoline series [5], leading to the selection of LMP400 (Indotecan), LMP776 (Indimitecan) and LMP744 for development.

The killing of cancer cells by camptothecins requires collisions between replication forks and the TOP1cc trapped by camptothecins (Figure 1, middle section). To excise TOP1cc, all eukaryotes (from yeast to humans) express the TOP1cc excision enzyme, TDP1 (tyrosyl DNA phosphodiesterase 1) (see middle section of the Figure 1). The ubiquitous presence of TDP1 is consistent with the fact that TOP1ccs readily form independently of camptothecin under a variety of circumstances that affect the canonical DNA structure including oxidative base lesions, base mismatches, abasic site, breaks and carcinogenic crosslinks [7]. Replication fork collisions generate replication stress, which engages the homology directed repair (HDR), ATR-CHEK1 and poly(ADP)ribose polymerase (PARP) pathways. HDR relies on the BRCA and Fanconi pathways. PARP, in addition to its regulatory effects on chromatin structure and repair nucleases, acts to recruit and activate TDP1 (bottom part of the Figure 1). In parallel with the repair pathways, cells can also activate the death pathway driven by p53 (TP53) and Schlafen 11 (SLFN11) [8] (bottom left of the Figure 1).

While the phase I clinical trials were ongoing with LMP400 and LMP776 [9], we initiated a randomized phase I trial of three novel indenoisoquinoline TOP1 inhibitors in the NCI Comparative Oncology Consortium (COP) (https://ccr.cancer.gov/Comparative-Oncology-Program) [10]. The Comparative Oncology Trials Consortium (COTC) is an active network of academic comparative oncology centers, which currently include 22 sites, centrally managed by the NIH-NCI-Center for Cancer Research’s COP. It functions to design and execute clinical trials in dogs with cancer to assess novel therapies. The goal of this effort is to answer biological questions geared to inform the development path of these agents for future use in human cancer patients. Trials conducted by the COTC are pharmacokinetically and pharmacodynamically rich with the product of this work directly integrated into the design of current human phase I and II clinical trials. Dogs with lymphoma were enrolled with the goal of comparing the potential antitumor activity of the two indenoisoquinoline in human clinical trials (LMP400 and LMP776) and of a 3^rd^, LMP744, which had been extensively studied preclinically but had not been initially selected for human trials because it was less active than LMP400 and LMP776 in murine xenograft models. The reported COP trial established their maximal tolerated dose, toxicity, blood and tumor pharmacokinetics and biomarkers of target engagement in the tumors and blood (γH2AX and TOP1 levels; middle right in the Figure 1) [10]. It confirmed that the two compounds (LMP400 – indotecan and LMP776 – indimitecan) initially selected and undergoing clinical trials in humans were active. Unexpectedly, the lymphoma dog trial revealed that LMP744 was even more active than LMP400 and LMP776; notably at doses well below its MTD. The potent activity of LMP744 was explained by its outstanding tumor accumulation and retention. Following the COP dog trial, LMP744 has entered into phase I clinical trial.
